# Multiple Myeloma in a Young Adult with Renal Involvement

**DOI:** 10.1002/ccr3.6986

**Published:** 2023-02-24

**Authors:** Meriam. Hajji, Samia. Barbouch, Rim. Goucha, Ezzeddine. Abderrahim

**Affiliations:** ^1^ Department of Medecine A Charles Nicolle Hospital Tunis Tunisia; ^2^ Laboratory of renal pathology LR00SP01 Tunis Tunisia; ^3^ Faculty of Medecine of Tunis Tunis El Manar University Tunis Tunisia

**Keywords:** cast nephropathy, early‐onset multiple myeloma, prognosis, renal biopsy, survival, treatment response

## Abstract

Multiple myeloma (MM) results from malignant plasma cell disorder. It represents approximately 10% of hematological malignancies and it is typically diagnosed in the elderly with a median age of 70 years and has a steep increase in incidence with advancing age. N Engl J Med. 2004, 1860; Clin Interv Aging. 2020, 619. The incidence of MM has been increasing over time, mostly due to population aging. Mayo Clin Proc. 2010, 225 However, certain MMs are diagnosed at young age even under 40 years old (2%). Leuk Lymphoma. 1998, 493; Blood. 2010, 5501. We report a case of a MM in a thirty‐four‐year‐old woman whose circumstance of discovery was acute kidney failure.

## Graphical abstract

(A)Trichrome stain: Intratubular polychromatophilic cylinder with cellular reaction all around (×200). (B) Trichrome stain: Presence of giant cells (×400). (C) Trichrome stain: Optically normal glomeruli (×200). (D) Direct Immunofluorescence: Absence of Ig deposits but presence of lambda on tubular basement membranes and cylinders.

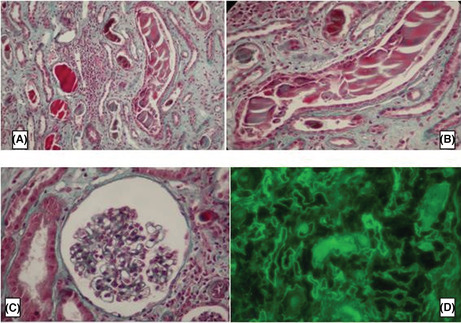

## 1 OBSERVATION

Ms. S.W, 34, with no medical history, was admitted for investigation of advanced renal failure. On admission, she was asthenic and complained of headache and gastric pain. On examination, she weighed 62 kg, she had normal blood pressure, correct hydration, and no abnormalities on cardiopulmonary auscultations. she had a preserved diuresis (1 L). The urine dipstick showed proteinuria: + and no hematuria.

**FIGURE 1:**
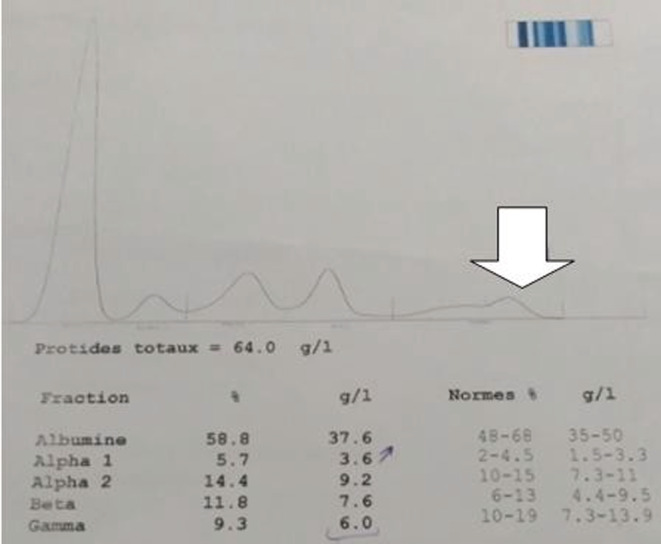
Monoclonal gamma peak at proteins electrophoresis in our patient.

**FIGURE 2:**
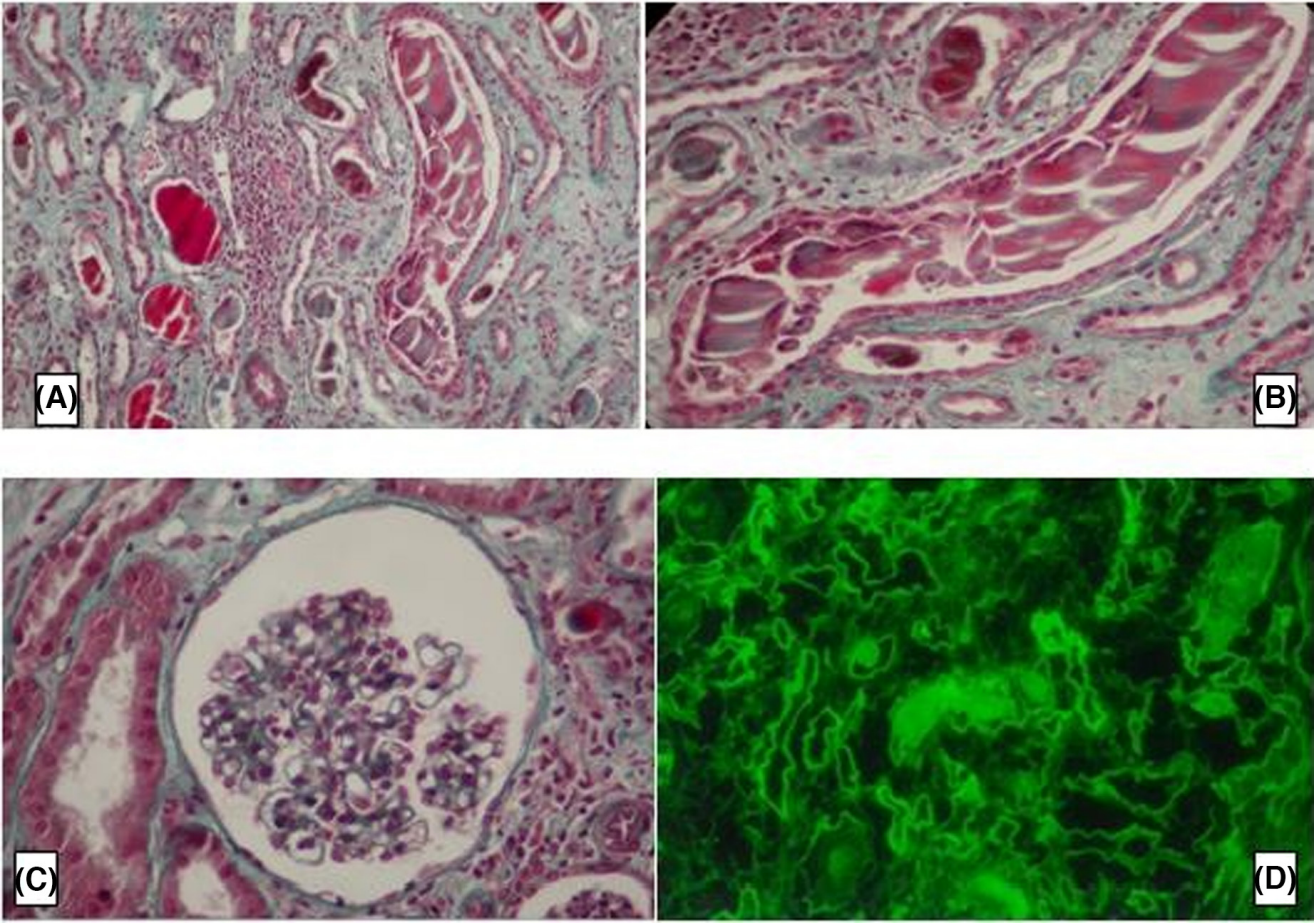
Renal biopsy (A) Trichrome stain: Intra tubular polychromatophilic cylinder with cellular reaction all around (magnification 200×) (B) Trichrome stain—presence of giant cells with (magnification 400×) (C) Trichrome stain—optically normal glomeruli (magnification 200×) (D) Direct Immunofluorescence: absence of Ig deposits but the presence of lambda on tubular basement membranes and the cylinders.

**FIGURE 3:**
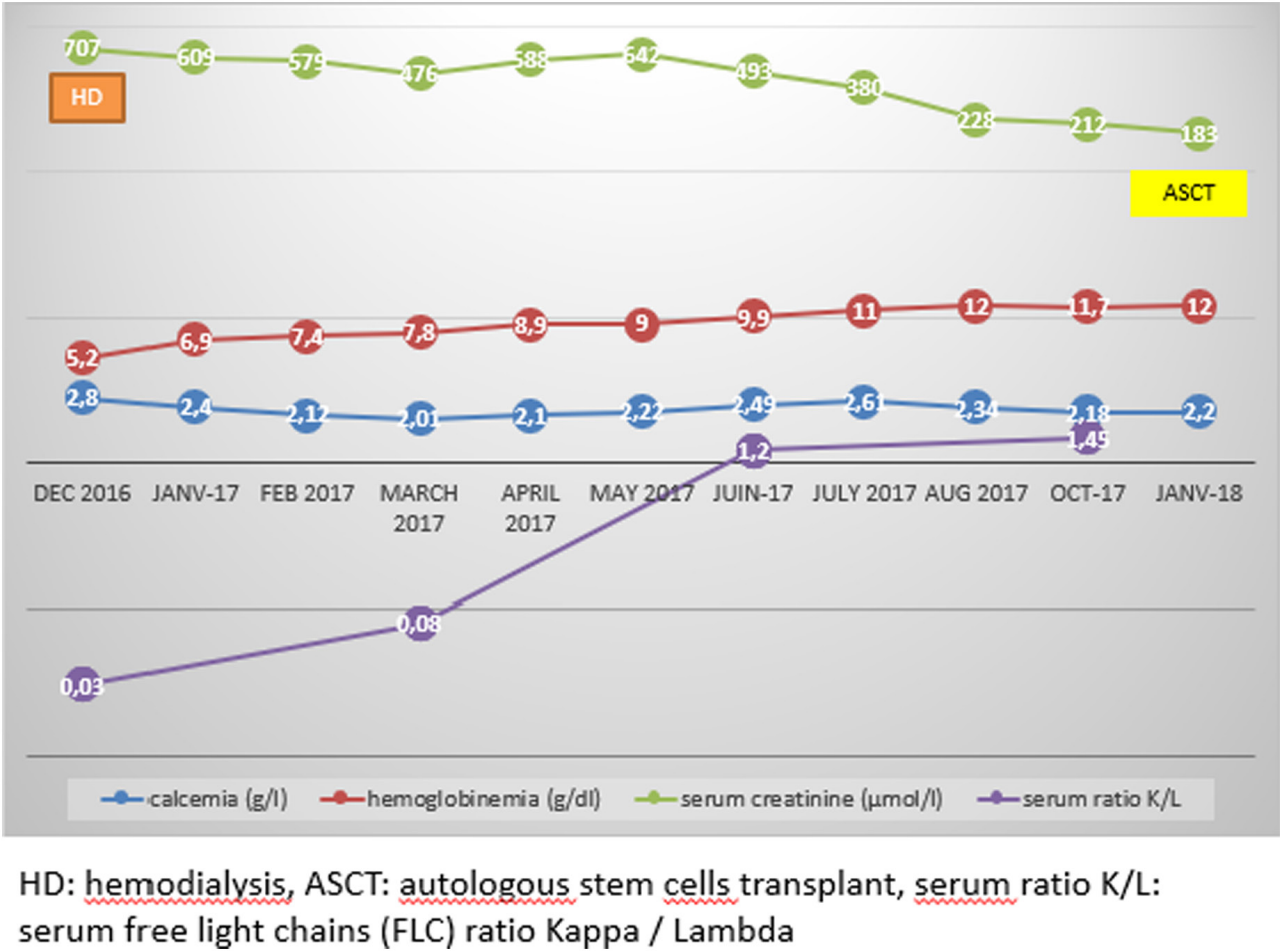
Kinetics of the various biological parameters throughout the follow‐up and chemotherapy treatment in our patient.

At biological assessment, she had serum creatinine at 707 μmol/L, normochromic normocytic anemia at 5.2 g/dL with direct positive IgG‐type Coombs test, serum calcium at 2.8 mmol/L and LDH level at 488 mmol/L. At protein electrophoresis: monoclonal peak in gamma position. Twenty‐four‐hour proteinuria dosage was 7 g/24 H, in contrast with low proteinuria with labstix. the blood ionogram showed a natremia at 140 mmol/L, a kalemia at 5.6 mmol/L and a chloremia at 110 mmol/L. At serum immunoelectrophoretic profile, the presence of a monoclonal gammopathy type Ig G Lambda (Figure 1). The serum‐free light chains (FLC) ratio kappa/lambda was equal to 0.03. Urine immunoelectrophoresis showed positive urinary monoclonal protein (free lambda chain); The ratio of kappa to lambda chains in urine was 0.017. On the myelogram: 32% plasmacytosis. On the kidney ultrasound, she had two well‐differentiated normalized kidneys. She had a renal biopsy, which showed an aspect of myelomatous tubulopathy (Figure 2). The spinal MRI was without abnormalities. CRAB criteria^6^ in our patient, involved hypercalcemia, renal failure, and anemia but no bone damage. The retained diagnosis in our patient was: MM with IgG Lambda class III of Salmon and Durie.^7^ R‐ISS prognostic factors^8^ included respectively serum albumin (37.6 g/L), serum β2 microglobulin (29.4 mg/L), and LDH level (488 mmol/L). Our patient was detected to have t (4:14) at Interphase fluorescence in situ hybridization (iFISH). Therefore, our patient was staged R‐ISS II. For the symptomatic treatment component, she had erythropoietin analogs; she required a total of 3 sessions of conventional hemodialysis with blood transfusion, notably before the kidney biopsy. When treating the causative disease, she received courses of Bortezomib (1.3 mg/m2) –Thalidomide (100 mg/day) –Dexamethasone (40 mg/day) according to the schedule of 6 cycles of 21 days each. Our patient presented a favorable clinical and biological evolution with an improvement in renal function (creatinine 212 pmol/l), anemia (Hb 12 g/dl), gamma globulin at 10.5 without a peak and seric free light chains ratio (kappa = 63, Lambda = 43, kappa/lambda = 1.45) after at least 1 year of admission to our department (Figure 3). The disease status in pre‐transplant was complete remission. On January 2018, she received an autologous stem cells transplant (ASCT) after conditioning with Bortezomib and Melphalan. Since then, she has evolved favorably and her renal function was stable (last serum creatinine in March 2021: 192 μmol/L).

## 2 DISCUSSION

Characteristics and prognosis of young patients with MM are not well known, and available literature comes from case reports and small series.^9^, ^10^, ^11^, ^12^, ^13^, ^14^, ^15^, ^16^ Hewell et al. have described the first documented cases of young patients with MM and reported a frequency of 1%.^9^ Blade et al. reviewed the records of 3278 patients treated for MM at the Mayo clinic between 1956 and 1992 and concluded that the incidence of MM in patients younger than 40 and 30 years was 2.2% and 0.3%, respectively.^10^ The clinical and biological characteristics of MM among young patients are as the same as in elderly patients in most series, there seem to be some specific aspects. A multicentric study found that men are more affected than women (sex ratio: 2/1).^17^ Higher incidence of the ISS 1 disease in younger patients with MM was also reported.^11^, ^13^, ^17^ In this context, some studies demonstrated that the prognosis in individuals diagnosed with MM before 40 years of age may be better than in older MM patients.^10^, ^14^ Several hypotheses have been ahead such as the lack of comorbidities in this population and the eligibility for ASCT. Another finding is that the presence of an elevated serum concentrations of LDH.^13^, ^14^ However, it is still unclear if the elevated level of LDH can be considered a specific feature of early‐age MM, however, it was mentioned in our patient. Usha et al. reported 14 cases of MM in patients <40 years old out of 178 cases, 10 of them had an IgG myeloma as was noticed in our patient.^18^ The second particularity of our observation is that renal impairment was not only part of the clinical presentation but also revealing of MM diagnosis. The pathogenesis of renal injury in MM is multifactorial. Excess FLC filtration and overburdening of proximal resorptive capacity, with subsequent uromodulin interactions in distal tubules, may precipitate cast formation, inducing tubulointerstitial damage and fibrogenesis.^19^ Cast nephropathy is the commonest cause of renal injury in MM, followed by hypercalcemia.^20^ Monoclonal proteins deposit along tubular basement membranes leading to a tubulointerstitial nephropathy, as it was the case in our patient. Few studies have investigated the most common types of MM in the young population but it has been reported that light‐chain disease (LCD) patients were slightly younger than IgA and IgG patients but older than IgD patients.^21^ As for our patients, It was a MM with IgG Lambda. The Kidney Disease Improving Global Outcomes (KDIGO) clinical practice guidelines for Acute kidney injury (AKI) recommend biopsy “if the cause of AKI is not clear after careful evaluation, especially in patients in whom prerenal and postrenal causes of AKI have been excluded, and the cause of intrinsic AKI is unclear. It is particularly useful when clinical assessment, urinalysis, and laboratory investigation suggest diagnoses other than sepsis, or ischemic or nephrotoxic injury”^22^ Renal biopsy is the key to confirm the association between a monoclonal protein and kidney disease. The spectrum of renal lesions that are seen in patients with myeloma includes “myeloma kidney,” or cast nephropathy; light‐chain (AL) amyloidosis; monoclonal Ig deposition disease (MIDD); and, less frequently, cryoglobulinemic glomerulonephritis and proliferative glomerulonephritis.^23^ Autopsy studies in patients with myeloma found cast nephropathy in 30%–50%, light‐chain deposition disease in 2%–3%, and amyloidosis in 4%–5% of cases.^24^, ^25^ In one study,^24^ acute tubular necrosis was seen in 34% of cases. Renal biopsy is the key to confirm the association between a monoclonal protein and kidney disease. The incidence of hypercalcemia and renal failure was documented in 2 large retrospective analyses but, did not confirm that younger age at the diagnosis predisposed to hypercalcemia and renal failure in MM patients.^12^, ^13^ Renal injury is a pejorative predictor of overall survival and increases the risk of additional complications in MM patients.^26^ Early diagnosis and treatment are crucial to control and reverse renal failure. The use of new markers of renal failure in MM is a promising direction and should be considered in the future.^27^ In a large study of patients diagnosed with myeloma before age 40,^28^ at 5 years, relative survival compared with same age‐ and sex‐matched individuals was 83.5%, and estimated standardized mortality ratio was 69.9 confirming that MM dramatically shortens the survival of young patients despite an extended survival after diagnosis. So far, our patient has presented a good prognosis.

## 3 CONCLUSION

Little is known about the disease characteristics and prognosis of these patients. The diagnosis of myeloma may result from the workup of unexplained renal disease. This diagnosis must be considered even at a young age since it involves the patient's prognosis, especially in the presence of an unexplained renal failure which should lead to renal biopsy.

## AUTHOR CONTRIBUTIONS

The first author made substantial contributions to acquisition and interpretation of data and writing the manuscript. The second author has been involved in revising it critically for important intellectual content. The third author contributed through the examination of histopathological lesions in renal biopsy and providing the photos. The last author has given final approval for the version to be published.

## FUNDING INFORMATION

No funding to declare.

## CONFLICT OF INTEREST STATEMENT

None declared.

## ETHICAL APPROVAL

The patient has given his written informed consent to publish his case including publication of images.

## INFORMED CONSENT

Written informed consent was obtained from the patient.

## Data Availability

Data sharing is not applicable to this article as no datasets were generated or analyzed during the current study.
